# Potential domestication and tameness effects on prosocial behaviour in chickens

**DOI:** 10.1371/journal.pone.0287213

**Published:** 2023-06-23

**Authors:** Rebecca Oscarsson, Per Jensen

**Affiliations:** IFM Biology, AVIAN Behaviour Genomics and Physiology Group, Linköping University, Linköping, Sweden; University of Queensland, AUSTRALIA

## Abstract

Prosocial behaviour is pronounced in humans and prevalent in some non-human animals, however, the occurrence of the trait in chickens has not yet been investigated. Here, we studied the occurrence of prosociality in four different lines of adult female chickens. To explore the effects of domestication, chickens of the domesticated layer White Leghorn (WL) and the ancestral Red Junglefowl (RJF) were compared. Additionally, to explore the role of tameness, Red Junglefowl selected for high (RJF HF), or low (RJF LF) fear of humans were also studied. The hens were all tested in a prosocial choice task adapted from a previous study conducted on rats. Each individual was first trained to differentiate between a compartment where itself and a companion received food treats simultaneously (representing a prosocial choice), and one where only itself received the treat. Following training, each bird was tested in a free-choice set-up. No occurrence of prosociality was found at group level in any of the lines, however, our results suggest that the trait may occur in some individuals, and that domestication and increased tameness may have increased its prevalence, although alternative explanations such as side bias and social competition cannot be ruled out. Since this study is the first of its kind, further research is required to make any definite conclusions.

## Introduction

Social behaviours of a negative character (e.g., aggression), have traditionally been the main focus in research on social behaviour in domestic animals [1]. More neutral or positive relationships (e.g. affiliation and attachment) are more pronounced in stable social groups [1] and can be regarded as positive welfare indicators, hence, positive social behaviours should receive more focus in research. Prosocial behaviour, one type of positive social behaviours, has been described as “a social act from a donor to a recipient, with the likelihood that this act benefits the recipient, without necessarily precluding benefits to the donor” [1]. Prosociality seems to be an intrinsic trait in humans [2], still, the trait has not been studied so much in non-human animals.

It has been suggested that domestication increases prosocial behaviour [3, 4]. However, there is a lack of research on prosociality in animals that have been domesticated, especially regarding systematic comparative studies of domesticates and their wild counterpart. Domestication can be viewed as a process whereby animals adapt to a life under human supervision [5]. This process gives rise to the domesticated phenotype, including changes in, e.g., appearance, physiology and behaviour [6, 7]. Chickens were domesticated about 8–9000 years ago from the ancestral Red junglefowl [8, 9], a highly social bird living in life-long family flocks [10]. Such a long-lasting and stable social lifestyle could be associated with prosociality, but to the best of our knowledge, no research has been conducted on that in this species.

Play behaviour has been suggested to be an example of prosocial behaviour [3]. A playful individual could promote the spread of positive welfare in groups [11], and thereby benefit conspecifics. A higher frequency of play has for instance been found in domesticated rats [12] and guinea pigs [13], when compared to their wild ancestors. Recently, we found a similar trend in chickens. Young domesticated White leghorn chicks were found to display a significantly higher amount of total play when compared to Red Junglefowl chicks of the same age [14].

Tameness has been hypothesized to be the primary selected trait that drives the domesticated phenotype [15]. To model early phases of domestication, we have selected Red Junglefowl for high and low fear of humans for 12 generations. From previous studies, we know that this selection for increased tameness results in trait alteration associated with the domesticated phenotype, e.g., increased body size, higher feed efficiency and production of larger eggs [16, 17]. Additionally, intra-specific social behaviour is affected as a correlated side effect of this selection [18], suggesting the possibility that increased tameness caused modifications of social behaviour early on during domestication. Here, we therefore attempted to assess prosociality in both ancestral Red Junglefowl versus domesticated chickens, and in high versus low fear Red Junglefowl in order to elucidate possible effects of tameness and domestication.

One test that has been used to study prosociality in non-human animals is the prosocial choice task. For example, Hernandez-Lallement and colleagues [19] performed a version of this test on rats. The rats were trained and tested in pairs in a double T-maze task, where one was used as actor, and the other partner. Actors chose between two alternatives differing in whether a reward was delivered to the actor only or to both the actor and the partner. The actor rats were found to choose “both rewarded” at levels above chance, indicating presence of prosocial behaviour. Prosociality is also present in birds. For example, a different version of the prosocial choice task was recently conducted on domestic canaries [20]. Here, the actor chose between three alternatives differing in whether a reward was delivered only to the actor, to both the actor and the recipient, or to neither of the two paired individuals. Several factors were found to impact the level at which prosociality was displayed, however, the actor canaries overall acted highly prosocial.

The aim of this project was to explore whether chickens will choose to act beneficial to other conspecifics in a choice test adapted from the one used by Hernandez-Lallement and colleagues [19] in rats. Additionally, we wanted to test whether this behaviour has changed during the domestication process, and therefore, domesticated chickens and wild Red Junglefowl were compared. Moreover, to explore the role of tameness, Red Junglefowl selected for high or low fear of humans were also studied.

## Materials and methods

### Ethical statement

This study was conducted at the University of Linköping, Sweden. The study was approved by the Linköping Council for Ethical Licensing of Animal Experiments, license number 14916–2018.

### Animals and housing

Females of two different breeds were used in this study, the domesticated layer White Leghorn (WL = 14) and the ancestral Red Junglefowl (RJF). Three different lines of Red Junglefowl were used: hens from an unselected line (RJF, n = 16), hens selected during 12 generations for low fear of humans (RJF LF, n = 7) or high fear of humans (RJF HF, n = 17). The sample sizes were unbalanced between the breeds, since we included all animals available from each group in the lab at the time of the experiment. The WL birds were from a non-commercial population, while the unselected RJF originated from a zoo population, both kept in our lab since 1998 (for detailed information about the background of the RJF and the WL, see [21]). The selected lines originated from an outbred group of two different zoo populations and had been selected based on fear score in a fear-of-human test carried out in every generation (for detailed information about the breeding and selection program, see [22]). At the start of testing, the RJF were 101 weeks, and the WL 91 weeks, whereas both the RJF LF and RJF HF were 43 weeks. All birds were bred, incubated, hatched, and housed under similar conditions at the University of Linköping, Sweden. From hatch until six week of age the birds were kept in the hatchery at the university campus, in pens ranging from 1×1 m– 2×3 m dependent on age and number of animals. Thereafter they were transferred to the breeding unit where they were kept in remodelled aviary systems (3×3×3 m for each group of 40–50 birds) with ad lib access to water, feed and perches at different levels, and with access to an outdoor range the same size as the pen.

Throughout the experimental period (two and a half days for each individual), the hens were temporarily housed in pairs inside the testing room, in cages (L×W×H: 0.74×1.21×0.82 m). The temporary housing cages had solid floor and were provided with wood chips, a perch, and feed and water ad lib. The animals were kept on a 12:12 h dark:light schedule throughout the experiment.

### Experimental setup

**Fig 1:**
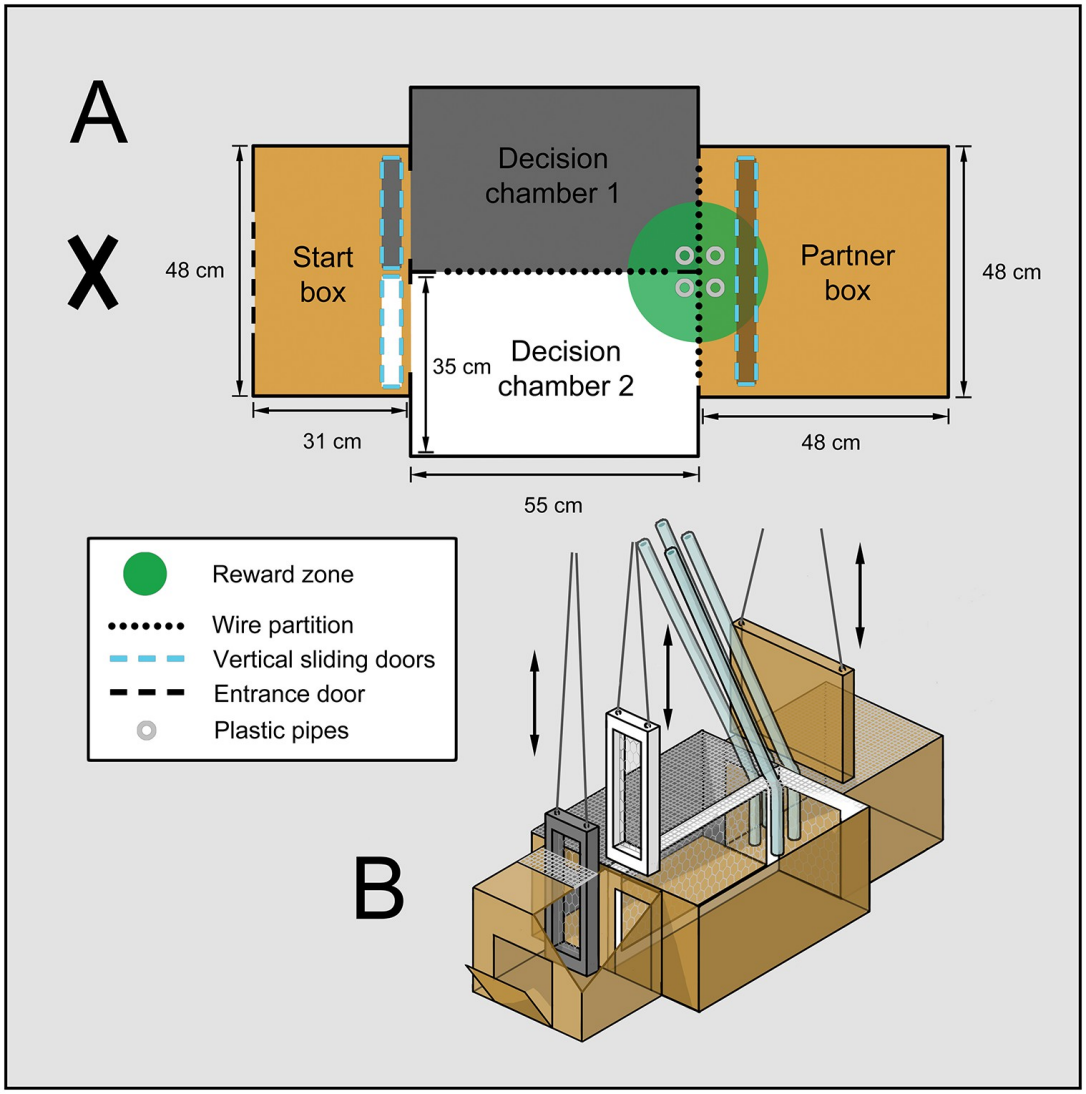
Experimental setup. **(A)** schematic drawing of the two-chamber test arena viewed from above. The arena consisted of a start box with two independent vertical sliding doors that each led to a decision chamber. The left door was grey, and the right door was white, the same colour as the decision chamber it led to. Opposite the start box, on the other side of the decision chambers, was the partner box. The decision chambers and the partner box were separated with wire mesh. The partner box was closed off by an opaque vertical sliding door when visibility had to be prevented. The start box was opaque, except for the part of the ceiling facing the decision chambers, which consisted of wire mesh, to allow some light entrance. The decision chambers and the partner box were covered with wire mesh. Rewards were provided through plastic pipes. Operation of all three doors and the delivery of rewards was all carried out from the experimenter position (marked by an X). **(B)** 3D illustration of the test arena setup.

The hens (hereafter: actors) had to choose between two options resulting in either only a reward for themselves (“own reward” OR) or a reward to both itself and a partner bird (“both reward” BR). The experiments were conducted in a two-chamber test arena (Fig 1). The test arena consisted of four compartments; a start box (L×W×H: 0.31×0.48×0.41 m) with two connecting decision chambers (L×W×H: 0.55×0.35×0.41 m), both accessible via separate vertical sliding doors, which consisted of a wooden frame and wire mesh. Opposite the start box, on the other side of the decision chambers, was the fourth compartment (L×W×H: 0.48×0.48×0.41 m), in which a partner was placed. The start box was opaque, except for to the part facing the decision chamber, where it consisted of wire mesh. The opaque covering blocked visibility of the experimenter to the actor when it was inside the start box, while the wire mesh still allowed light entrance. To further help the distinction between the decision chambers, the left one had grey walls, and the right one white. Also, the frame of the chamber doors was of the same colour as the chamber it accessed. The two decision chambers, as well as the partner box and the decision chambers, were separated with wire mesh. At times when visibility had to be prevented, the partner box was closed off by an opaque vertical sliding door. To avoid visual contact with other birds in the test room, white curtains covered the cages and the test arena.

One and the same experimenter performed all training and testing alone. To not influence the actors’ choices, each step of each test was carried out from the same spot, behind the start box and out of sight of the actor. The three sliding doors were opened and closed with strings, operated by the experimenter. Live mealworms were used as a reward. The rewards were provided through plastic pipes reaching from the experimenter position down to the reward location (Fig 1A).

### Habituation and forced trial sessions

The total test procedure took three days for one pair of hens. The same two hens went through all the steps together and took turns having the role as actor and partner. Each test procedure consisted of one day of habituation followed by a first training session with forced trials. The first day consisted of a habituation period of 30 minutes, where the birds spent 15 minutes in each role (actor and partner). At this stage, all three doors, the two netted decision chamber doors and the opaque partner box door, were open. The hen on the actors’ side could move freely between the start box and the left and right decision chamber. The final ten minutes of each 15 minutes session, all three doors were closed and opened three times, approximately three minutes apart as part of the habituation process. After the habituation session, the first forced trial session was carried out. Here, each hen went through a series of 20 forced choice trials, ten in each chamber. During these trials, only the door to one of the chambers was opened, and the treat was delivered to the actor only in one of them and to both actor and partner in the other. To facilitate the finding of the reward location, the treats were at first delivered right after the actors had entered a decision chamber and the door had been closed behind them. As the hens were then most often facing forward, they could easily detect the reward being delivered. The partner was visible to the actor the entire time. The goal of the first forced trial session was that the birds should find the reward location and start associating the decision chambers with the two reward conditions.

The second day, two forced trial sessions were conducted, during which each hen went through a series of 30 forced choice trials (15 to each decision chamber) on two separate occasions (in total 60 forced trials). The partner was visible to the actor the entire time. The goal of the second and third forced trial session was to strengthen the birds’ associations of the compartments with the two reward conditions. To avoid the actor’s preference being influenced by asymmetrically timed reward delivery, a fixed reward delivery time of 10 seconds after decision chamber entrance was introduced in the second training session on day two.

The third day, a shorter forced trial session was carried out before starting the test trials. Here, a final element was introduced. After two forced choice trials (one to each decision chamber) with the partner door open, another eight forced trials (four to each decision chamber) were conducted with the partner door closed as the actor was let out of the start box, followed by being opened before the reward(s) was delivered. The purpose of this was to force the actor to make its choice based on prior association of each chamber with either OR or BR, regardless of the position of the partner in the partner box.

Which chamber that was associated with OR and which with BR was systematically altered between individuals within each breed/line of hens. However, for each pair the same side was always associated with OR and BR respectively.

### Test sessions

Following the habituation and training sessions, the actual tests took place. After two forced choice trials (one to each decision chamber) with the partner door open, each hen was exposed to 40 free choice trials with the partner door closed when the actor chose compartment, followed by being opened before the reward was delivered. One hen went through all 40 free choice trials before the roles were switched and the partner hen was exposed to 40 trials. To make sure the actor noticed the partner’s reward during a BR choice, the mealworms were delivered nine and ten seconds respectively to the partner and actor after the partner door was opened. Between successive trials, 5 s was allowed to pass during which the actor was in the start box with full visual access to each reward chamber.

### Data analysis

We assessed whether subjects preferred the prosocial (BR) side more than the non-prosocial (OR) side both at the group and individual level. To assess any possible group level differences between breeds with respect to number of BR choices, a Generalized Linear Model was used with probability distribution “poisson” and link function “log”, using breed/line and the colour of the BR chamber as factors (to control for possible side bias). Differences were considered significant when P < 0.05. The GzLM was carried out in SPSS 28.0.1.

To assess preferences above chance level at the individual level, we performed binomial tests. The subjects were considered to have a significant preference once the probability of the number of BR choices compared to the null hypothesis of 50% choices was below p < 0.05.

## Results

**Fig 2:**
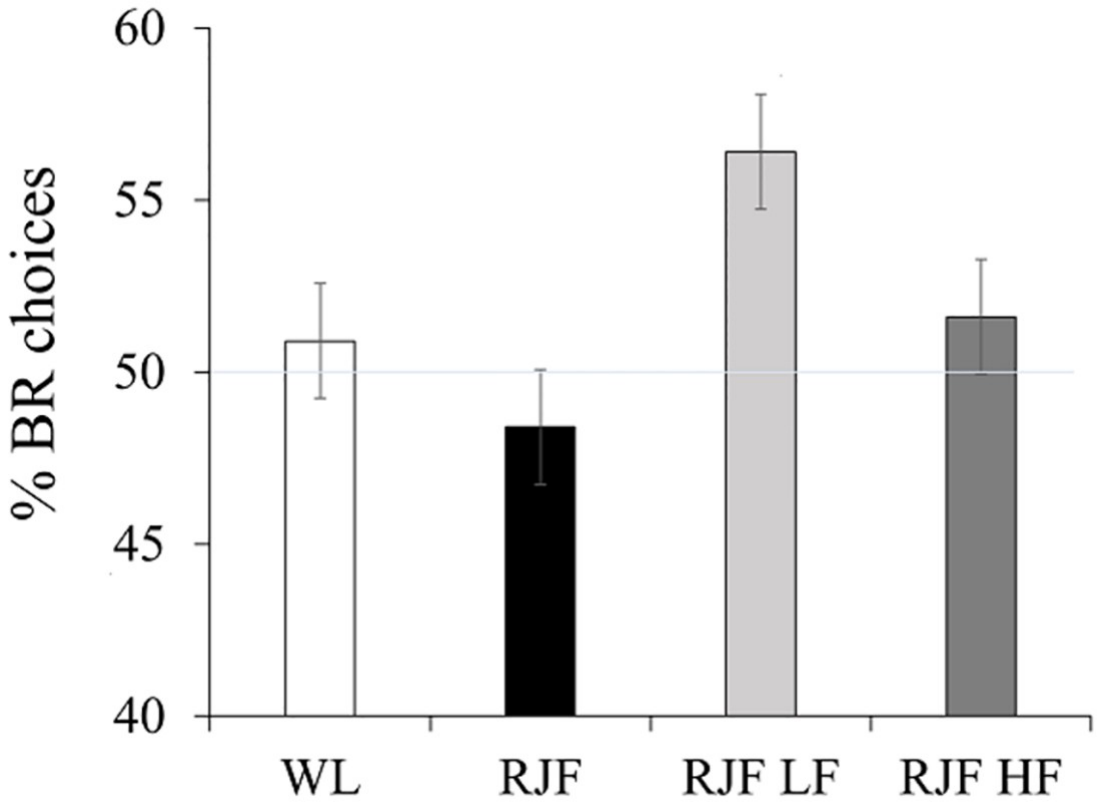
Average percentage of BR choices (+/- SEM) for each group of birds. The tinted line marks the 50-percentage limit. Notice that the y-axis starts at 40 percent.

The average percentage of BR choices at the group level (for each breed/line) are illustrated in Fig 2. There was no significant difference in prosociality, nor any side bias found, among the four groups (prosociality: χ^2^ = 1.838, df = 3, p = 0.607; side bias: χ^2^ = 0.562, df = 1, p = 0.454). However, there was a significant interaction between breed/line and the BR side (χ^2^ = 17.583, df = 3, p < 0.001), due to the fact that WL chose the grey compartment more often than the white, while the RJF HF chose the white compartment more often than the grey, regardless of whether it was associated with BR or OR.

Table 1 summarizes the choice behaviour of the individual animals. A total of 19 animals chose one chamber over the other above chance level. Four WL, one RJF, three RJF LF and three RJF HF showed a significant preference for the BR side (p < 0.05), while two WL, two RJF, one RJF LF and three RJF HF showed a significant preference for the OR side (p < 0.05).

**Table 1:** For each group, number of individuals that acted prosocial (BR) above chance level, number of individuals that acted asocial (OR) above chance level, number of individuals that showed no significant preference (No), and total number of individuals that were tested (Total).

Breed/line	BR	OR	No	Total
**WL**	4	2	8	14
**RJF**	1	2	13	16
**RJF LF**	3	1	3	7
**RJF HF**	3	3	11	17

Relative to the total number of individuals in each group, the WL (4/14; 28%) and the RJF LF (3/7; 43%) had the highest number of individuals preferring the BR side above chance level. Additionally, in these two groups, twice as many, or more, individuals showed a significant preference for the BR side, compared to the number of individuals showing a significant preference for the OR side.

Table 2 summarizes the choice behaviour within each pair of birds. In 16 pairs, at least one individual chose one chamber over the other above chance level. In three out of these 16 pairs, both individuals showed a significant preference for either chamber. In only one pair (RJF LF), both individuals showed a significant preference for the BR side. Out of the 11 individuals with a BR preference, six were tested as actors first and as partners second, and five were tested as partner first and as actor second.

**Table 2:** For each group, the table shows number of pairs which consisted of one individual acting prosocial and one asocial (BR/OR), number of pairs which consisted of two individuals acting prosocial (BR/BR), number of pairs which consisted of two individuals both acting asocial (OR/OR), number of pairs which consisted of one individual acting prosocial and one individual showing no significant preference (BR/No), number of pairs which consisted of one individual acting asocial and one individual showing no significant preference (OR/No), and number of pairs where neither individual showed a significant preference.

Breed/line	BR/OR	BR/BR	OR/OR	BR/No	OR/No	No/No
**WL**	0	0	0	4	2	1
**RJF**	0	0	0	1	2	5
**RJF LF**	0	1	0	1	1	1
**RJF HF**	2	0	0	1	1	4

## Discussion

We evaluated the occurrence of non-costly prosocial behaviour in four groups of hens, using a chicken version of a Prosocial Choice Task. No significant prosociality was found at group level, however, Red Junglefowl selected for low fear of humans (RJF LF) showed the numerically highest proportion of prosocial choices. Furthermore, the RJF LF and the White Leghorns (WL) had the highest number of individuals acting significantly prosocial relative to the total number of individuals tested. Although different interpretations of these results are possible, they indicate that prosocial behaviour may occur in some individuals and might also possibly indicate that domestication and increased tameness has influenced prosociality in chickens, in the sense that the trait has become more prevalent. Unfortunately, considering the smaller sample size of the RJF LF, the findings of this group is unavoidably weaker than that of the WL.

Although we could not confirm any breed differences in prosociality on a group level, there were individuals acting significantly prosocial in all tested groups. The lack of group level effects was mainly due to a lack of choice of any of the two options in the majority of individuals, but also that in all groups there were some individuals that acted significantly “selfish” by mostly choosing the OR option. Nevertheless, in all four groups some birds acted prosocial as judged by their significantly higher choice of the BR option. This indicates that prosociality may be a feature of specific individuals or specific pairs of birds. However, there is also possibility that the performance at chance found at the group level is not due to a lack of prosocial tendencies, but rather, at least partly, a result of the hen’s inability to learn the association between the chambers. This explanation may most likely apply to the individuals that showed no preference for either chamber.

Besides the potential inability to distinguish between the outcomes of the chambers, there is the possibility that the test set up might have stimulated competitive behaviour in the actor hens. For instance, despite being separated by a transparent partitioning, domestic chicks have been found to forage quicker when a conspecific is visible [23], suggesting either that social facilitation or perceiving possible competition stimulates feeding. Hence, we cannot exclude that the actor hens perceived the test situation as competitive. A motivation to avoid such perceived competition could potentially have been the underlying motivation of the individuals acting asocial (choosing only own reward). Hence, the prosocial individuals were perhaps the ones that were able to both learn the association of each chamber and also comprehend that the partner hen could not access their own food reward. Also conceivable is that the hens with a BR preference chose predominantly prosocially to avoid threatening behaviour displayed by their partner when only the actor got rewarded, although this was not indicated by any observations during the tests.

The ancestral Red Junglefowl is a highly social species, where the birds mostly spend the largest part of their lives within one and the same family group, and given the opportunity, domesticated chickens have a similar social system [24]. Such long-lasting relationships form the foundation for complex social networks, including dominance hierarchies and inter-individual affiliations [1, 25]. The fact that some birds in our experiment showed what could be prosocial behaviour, while others did the opposite, may be a result of inter-individual relationships that we were not able to account for. The hens were picked and paired randomly from the home pens. Each pair of hens were therefore familiar to one another, however, apart from that, we are unaware of their relationships. Chickens in a social group are known to establish a dominance hierarchy amongst themselves. The hens in the current study were housed together from hatch, so a social hierarchy was with certainty established since long. Since the birds were selected randomly, it is possible that dominance relations may have affected their choices in the test situation. Most individuals with a significant side preference had a partner that showed no preference. Hence, in all pairs except one (RJF LF), the paired individuals did not match each other in their choices. This could possibly indicate a difference in dominance status. Personality is another likely impacting factor. Chickens do possess individual personalities and can therefore be individually unique in their behaviour [26]. In a study of domestic canaries, personality did seem to affect level of prosociality [20], where proactive individuals learnt faster and were more prosocial than slower learning, reactive individuals. It is possible that not only relationship status but also personality differences affected the hens’ choices, but since we lack information on these aspects, this remains speculations. For future studies, it should be considered to additionally assess both dominance relationships and personality traits in the animals tested. This added assessment could potentially lead to different results regarding prosociality. Furthermore, there is a chance that some hens developed a side preference for either chamber, and if so, that this influenced their choices. Hens have previously been found to develop a side preference in a two-choice task [27] and this was further linked to personality characteristics, where more fearful and stressed hens showed a stronger side bias. In future studies, it would be advisable to vary the sides of the OR and BR compartment between trials to achieve a balanced setup to control for such possible side bias.

It has been suggested that domestication increases prosocial behaviour in different species [3, 4]. The reasons for this assumption are not completely clear, but one could imagine that excess of resources (food, shelter) and reduced predation risks, two important aspects of domestication, would benefit the evolution of less selfish acts. Similar to the present experiment, Dale and colleagues [28] also adapted the method of Hernandez-Lallement et al. [19] (who studied mice) and piloted the location choice task when comparing the occurrence of prosocial behaviour in dogs and wolves. Contradicting their hypotheses, neither species, apart from one single wolf that was acting prosocial, were found to display any preference between the choices. However, as acknowledged by the authors, only a small number of animals participated, and a larger sample size may have resulted in a different outcome. However, supporting these results, wolves but not dogs acted prosocial when compared in a touch screen task [28]. Hence, the two studies by Dale and colleagues [28, 29] do not provide any strong support for the argument that prosocial behaviour increased during domestication. However, it has also been suggested that play behaviour can be regarded as a prosocial activity, and in many domestic animals, play is more abundant than in the ancestors [3, 4]. We recently showed that young, domesticated chickens play significantly more than Red Junglefowl, possibly supporting the suggestion that aspects of prosocial behaviour may increase in domesticates [14].

As should be clear from the discussion above, individual relationships are likely to affect the outcome of the choice test used here. We attempted to reduce this factor by allowing the actor to make its choice when the partner was not visible, and by doing so, we also avoided that the current location of the partner inside the partner box impacted the actors’ decisions. However, what was not possible to control for was the position of the actor inside the start box. This would have required restraining the animals somehow, which was considered neither practically feasible nor beneficial to the study. Hence, it is possible that the choice of the actor was partly affected by which compartment it was facing when the doors were opened. To avoid influence, the experimenter was not visible to the actor hen when inside the start box and therefore the actor hen was also not visible to the experimenter, hence, its position could not be assessed. To further avoid influence by the experimenter, a set time was applied between trials and from chamber entrance until reward delivery. In future studies, video recording all trials should be considered to enable the confirmation afterwards that criteria like these were indeed met.

Test order did not seem to have an effect. The two hens in each pair were tested one after another. About half of the individuals with a prosocial preference were tested as actor first, and about half as actor second. The experience of being partner first could potentially result in a primed preference for the BR side when later tested as actor. However, if test order did not have an effect, then it is likely that by chance, half of the prosocial individuals were tested as actor first and half as actor second. Ideally, to completely dismiss any effects of test order, only one hen in each pair would have been tested as actor. This however would have required that many more birds and was therefore not an option for this study.

Age is another possibly impacting factor. Although all were sexually mature, the chickens tested in this study were of slightly different ages. In humans, inconsistent results are found regarding adult age difference on prosociality. Prosocial tendencies tend to increase with age in adulthood [30, 31], however, no effect of age has also been found [32]. Hence, age could potentially impact prosociality also in non-human animals, chickens included. However, it is important to note that within each main comparison, i.e., WL vs RJF and RJF HF vs RJF LF, the birds were of about the same age, so age would not confound those results.

In conclusion, to the best of our knowledge, this is the first study to investigate the occurrence of prosociality in chickens. Our results suggest that prosociality may occur in some chickens, or in some specific pair-relations. Furthermore, our results suggest that domestication and increased tameness may possibly have led to prosociality becoming more prevalent in chickens, although in this experiment we are not able to exclude possible effects of competition and side bias. In future experiments, this would need to be considered.

## Supporting information

S1 Data (XLSX)
